# Epidemiological profiles and outcomes of healthcare workers hospitalized for COVID-19 in five Sub-Saharan African countries: a cohort study

**DOI:** 10.12688/f1000research.150775.1

**Published:** 2024-06-18

**Authors:** Tamba Mina Millimouno, Fassou Mathias Grovogui, Karifa Kourouma, Shermarke Hassan, Ibrahima Kaba, Ibrahim Franklyn Kamara, Jerry-Jonas Mbasha, Timire Collins, Laura Merson, Alexandre Delamou

**Affiliations:** 1 Africa Center of Excellence for Prevention and Control of Communicable Diseases (CEA-PCMT), Gamal Abdel Nasser University of Conakry, Conakry, Guinea; 2 Centre National de Formation et de Recherche en Santé Rurale de Maferinyah, Forecariah, Guinea; 3 Public Health Department, Gamal Abdel Nasser University of Conakry, Conakry, Guinea; 4 Infectious Diseases Data Observatory, University of Oxford, Oxford, UK; 5 Center for Tropical Medicine and Global Health, Nuffield Department of Medicine, University of Oxford, Oxford, UK; 6 Reproductive Maternal Newborn Child and Adolescent Health Unit, Universal Health Coverage/Life Course Cluster, World Health Organization Country Office, Freetown, Sierra Leone; 7 World Health Organization, Africa Regional Office, Brazzaville, Congo; 8 International Union Against TB and Lung Disease (The Union), Paris, France; 9 ISARIC (International Severe Acute Respiratory and emerging Infections Consortium), Pandemic Sciences Institute, University of Oxford, Oxford, UK

**Keywords:** Pandemic, COVID-19, Healthcare workers, Epidemiological profiles, Outcomes, Sub-Saharan Africa

## Abstract

**Background:**

The COVID-19 pandemic placed immense strain on global health systems and healthcare workers (HCWs). This study aimed to analyze the epidemiological profiles and outcomes of HCWs hospitalized for COVID-19 across five sub-Saharan African countries.

**Methods:**

This was a cohort study using secondary data collected between January 30, 2020, and December 31, 2022, as part of the International Severe Acute Respiratory and emerging Infection Consortium study. The study population consisted of HCWs who were hospitalized due to clinically suspected or laboratory-confirmed SARS-CoV-2 infection. Demographic and clinical characteristics and case management were summarized using proportions or medians and interquartile ranges. Factors associated with risk of mortality among HCWs were assessed using a Cox proportional hazards model, adjusted for age and sex.

**Results:**

Findings showed that South Africa recorded a lower percentage (2.4%) of patients who were HCWs compared to Gambia, Ghana, Guinea, and Malawi. Overall, many HCWs were aged ≥50 years and the majority were females (66.8%). In three of the five countries, however, the majority of the HCWs were <39 years old and were males. Antibiotics were the most commonly used medical treatments across three countries (Ghana, 67.8%; Guinea, 97.3%; Malawi, 80%), while antimalarials were commonly used in Guinea (87.8%) and Ghana (30.4%). Guinea and South Africa reported deaths with case-fatality rates varying from 22% in March 2020 to 1.4% in February 2022. Risk factors for mortality included age over 50 years, hypertension, diabetes mellitus, and chronic kidney disease.

**Conclusions:**

Our study underscores the critical need for continuous protection and enhanced readiness for HCWs, particularly during epidemics and pandemics. Strengthening infection prevention and control measures and focusing on vulnerable groups such as older and female HCWs are essential for mitigating the impact of future health crises. Further research is required to fully comprehend the implications of these findings.

**List of abbreviations**
CFR: Case Fatality Rate
CI: Confidence Interval
COVID-19: Coronavirus Disease of 2019
EAG: Ethics Advisory Group
HCW: Healthcare Worker
HR: Hazard Ratio
IDDO: Infectious Diseases Data Observatory
IQR: Interquartile Range
ISARIC: International Severe Acute Respiratory and emerging Infections Consortium
MoH: Ministry of Health
PPE: Personal Protective Equipment
REDCap: Research Electronic Data Capture
SARS-CoV-2: Severe Acute Respiratory Syndrome Coronavirus 2
SORT IT: Structured Operational Research and Training Initiative
TB: Tuberculosis
WHO: World Health Organization

## Introduction

The COVID-19 pandemic burdened health systems and their healthcare workers (HCWs) globally. As of May 2021, the World Health Organization (WHO) reported 179,500 deaths among HCWs due to COVID-19. ^1^ However, HCWs are critical to global health security and their safety during public health emergencies benefits society as a whole. ^2^ Loss of HCWs due to death or ill health has socioeconomic costs to society and may be associated with an increase in secondary infections and excess deaths (e.g., maternal and child death) due to a shortage of skilled staff. ^2^ The fact that HCWs continue to be infected or are a source of infection during public health emergencies proves weaknesses in global preparedness efforts. ^3^^,^^4^

In high-income countries, several studies sought to understand the epidemiological characteristics and outcomes of HCWs infected with COVID-19. ^5^^–^^13^ Those studies reported that the proportion of HCWs infected with COVID-19 ranged between 2.7% and 12.9%. They revealed that infected HCWs were younger and mostly female, and recorded case fatality rates varying between 0.3% and 3.3%. HCWs working in COVID-19 isolation units and treatment centers also had a higher risk of being infected with SARS-CoV-2 than non-HWCs. ^14^

This problem was exacerbated in Sub-Saharan Africa, where there were major gaps in response capacity, including in human resources and protective equipment. As early as November 2020, Okeahalam et al. reported that there was a lack of qualified nursing personnel to help mitigate the effects of the COVID-19 pandemic. ^15^ An assessment of health system preparedness for the COVID-19 pandemic in Guinea in 2020 revealed inadequate preparedness of health facilities and services along with discrepancies in preparedness between public and private health sectors and between locations. ^16^ The study also reported that many HCWs felt unsafe at their workplace concerning workplace exposure to SARS-CoV-2. While data on epidemiological features and COVID-19-related mortality among HCWs have been documented in high-income countries, there is still limited information about this in Sub-Saharan Africa. Some studies highlighted similar patterns in terms of gender and age at risk of COVID-19 infection and variations in the magnitude of the disease among HCWs. ^17^^,^^18^ It is possible that different patterns exist across countries and regions in Sub-Saharan Africa due to various contextual factors such as the status of the health system and experience with previous epidemics. This means that there is still a knowledge gap as current studies do not provide a larger picture of epidemiological features and outcomes among HCWs who contracted COVID-19 in Sub-Saharan Africa. Hence, our study aimed to fill this existing knowledge gap and to inform pandemic preparedness efforts in Sub-Saharan countries.

The main objective of this study therefore was to analyze the epidemiological profiles and outcomes of HCWs hospitalized for COVID-19 in five countries in Sub-Saharan Africa from January 2020 to December 2022. Specifically, we described demographic and clinical characteristics on admission, the case management, and hospital exit outcomes among HCWs, and we assessed their risk factors for death.

## Methods

### Study design

We conducted a cohort study involving secondary data collected as part of the International Severe Acute Respiratory and emerging Infection Consortium (ISARIC).

### Setting

***General setting***

*Sub*- *Saharan Africa* covers 51 countries on the African continent that lie south of the Sahara. It had an estimated population of 1.2 billion inhabitants in 2023. ^19^ The first case of COVID-19 in Sub-Saharan Africa was reported on January 28, 2020 in Nigeria. ^20^ As of July 12, 2023, about 5.8 million confirmed COVID-19 cases in Sub-Saharan Africa had been reported to the WHO, representing 40% of all COVID-19 confirmed cases in Africa. ^21^ As of the same date, 86.5 thousand COVID-19 deaths in Sub-Saharan Africa were reported to WHO, representing 49% of COVID-19 deaths in Africa. ^21^

***Specific setting***

**Figure 1.**
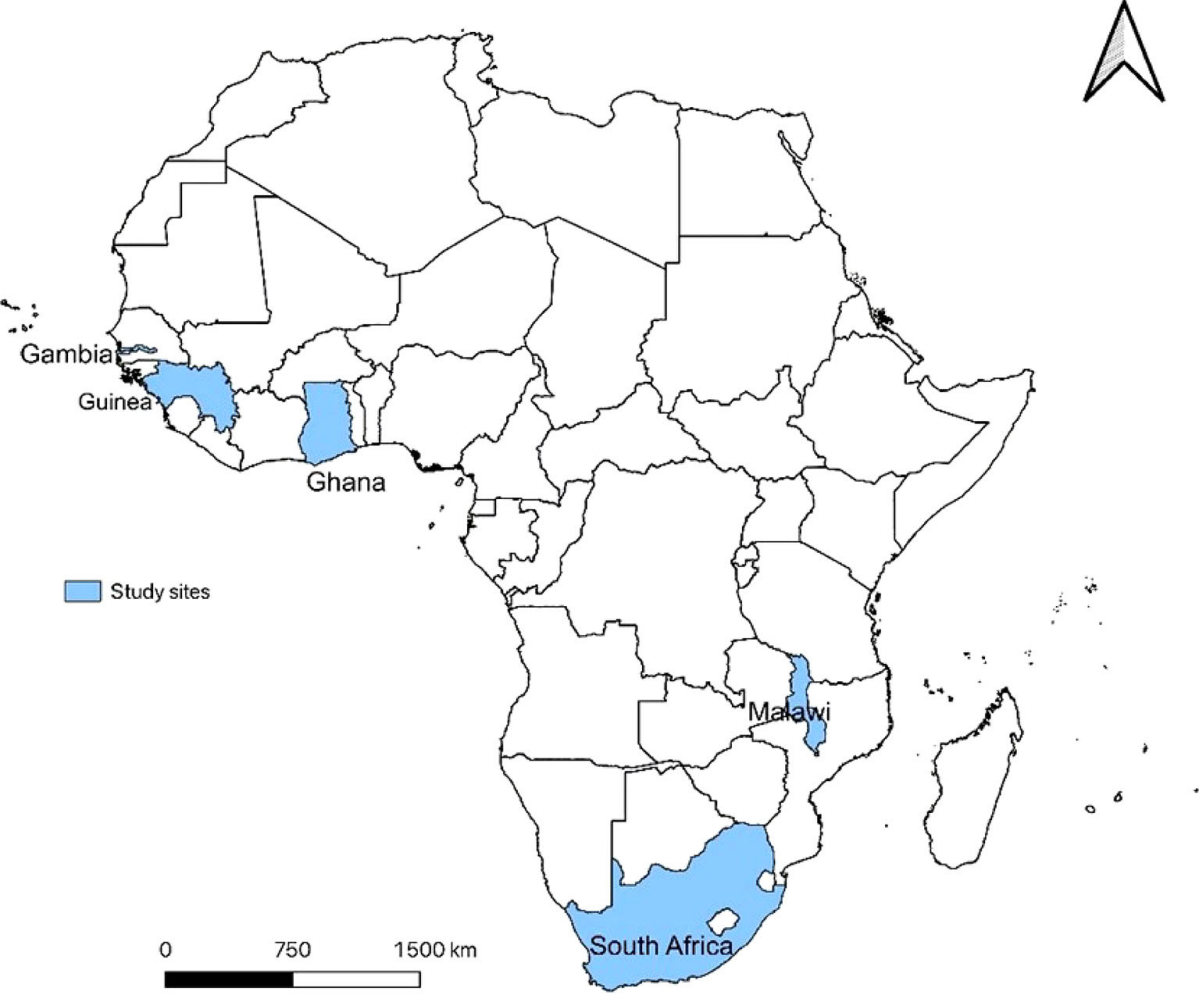
Countries included in the study on epidemiological profiles and outcomes of HCWs hospitalized for COVID-19 in Sub-Saharan Africa, from January 2020 to December 2022.

All Sub-Saharan countries that reported COVID-19 data to ISARIC, and where HCWs were affected by the disease based on the reported data, were considered for the current study. These included Guinea, Gambia, Ghana, Malawi, and South Africa ( Figure 1). COVID-19 preparedness and response were organized differently in these countries. In some countries, the response to the pandemic was led by an agency of the Ministry of Health (MoH), such as the National Agency for Health Security (Guinea), Ghana Health Service (Ghana), and in others they were led by the MoH itself (Gambia, Malawi, and South Africa). In the early phase of the pandemic, WHO set up guidelines with progressive updates for the clinical management of COVID-19, that were adopted by countries. ^22^

### Study population and period

The study population consisted of HCWs from Guinea, Gambia, Ghana, Malawi, and South Africa who were hospitalized due to clinically suspected or laboratory-confirmed SARS-CoV-2 infection between 30 January 2020 and 31 December 2022.

### Variables and source of data

Data had already been collected as part of the ISARIC study, an international prospective observational study on demographics, clinical features, and outcomes of patients admitted to hospitals with COVID-19. Data variables included demographic and clinical characteristics of HCWs on admission (age, gender, year of hospital admission, country of residence, diagnosis, duration of hospitalization and comorbidities), variables on case management during hospitalization (medical treatment received: agents acting on the renin-angiotensin system, antibacterials, non-steroidal anti-inflammatory and antirheumatic products, antimalarials, antimycotics, antivirals, and corticosteroids; and admission to intensive care unit), and hospital exit outcome variables (death).

### Data collection and validation

Data were collected as part of the ISARIC-WHO Clinical Characterisation Protocol contributing to a central repository at the University of Oxford, England. Participating sites used the ISARIC-WHO case report form to enter data onto a Research Electronic Data Capture ( REDCap, version 8.11.11, Vanderbilt University, Nashville, TN) database or used local databases before uploading to the central data repository ( Open Data Kit is a suitable open access alternative). Centrally collated data were wrangled and mapped to the structure and controlled terminologies of the Study Data Tabulation Model, version 1.7, Clinical Data Interchange Standards Consortium, Austin, TX) using Trifacta software ( OpenRefine is a suitable open access alternative https://openrefine.org/). The data collection, aggregation, curation and harmonization process have been described previously. ^23^

### Data analysis and statistics

We imported the datasets (in MS Excel format) into R software (R: The R Project for Statistical Computing), version 4.3.2. We summarized the demographic and clinical characteristics of HCWs and the case management of HCWs by country. Descriptive statistics were reported as proportions or medians with interquartile range (IQR). For the countries that recorded deaths, the monthly case-fatality rate (CFR) over time was shown visually in a line plot.

We assessed the association between demographic and clinical characteristics and the risk of mortality among HCWs using a Cox proportional hazards model, adjusted for age and sex. The strength of associations was measured using hazard ratios (HR) and their 95% confidence intervals (CI). The result was reported visually as a forest plot.

### Ethical considerations

Execution of the ISARIC-WHO Clinical Characterisation Protocol was approved by the WHO Ethics Review Committee (RPC571 and RPC572, 25 April 2013) and by local or national Ethics Committees for participating sites. Approvals included the Witwatersrand in South Africa as part of a national surveillance program (M160667), which represents the majority of the data. Written consent for patient data to be collected and used in research, was obtained or waived according to local norms determined by the responsible Ethics Committee. The data were collected using the ISARIC-WHO case report form. Arrangements surrounding the pooling, storage, curation and sharing of these data are covered by the Infectious Diseases Data Observatory (IDDO) Governance processes. Execution of this secondary analysis was approved by the Ethics Advisory Group (EAG) of the International Union against Tuberculosis and Lung Disease, Paris, France (EAG approval number 19/23 of 08/09/2023).

All data were deidentified and ensured of low risk for identification of individuals by a statistical disclosure process before sharing. Data were shared under a Data Access Agreement following approval from the IDDO Data Access Committee. The dataset used in the analysis will be destroyed by the study team following publication but remain available for access via the IDDO platform.

## Results

There were 482,457 patients hospitalized for COVID-19 in five Sub-Saharan African countries (Guinea, Gambia, Ghana, Malawi, and South Africa) who had information on HCW status. Overall, 11,421 out of 482,457 (2.4%) patients were HCWs. The percentage of patients who were HCWs was 4% in Ghana (99 out of 2,494), 3.3% in Guinea (76 out of 2,294), 17.5% in Gambia (7 out of 40), 2.9% in Malawi (10 out of 340) and 2.4% in South Africa (11,229 out of 477,289).

### Patients’ demographic and clinical characteristics and treatment comorbidities

Table 1 depicts the patients’ demographic and clinical characteristics. Overall, most COVID-19-affected HCWs were aged ≥50 years with a median age equal to 50 years (IQR: 39-59); however, patients were mostly younger (<39 years) in Gambia (42.9%), Ghana (67.7%), and Guinea (51.3%). Overall, patients were mainly female (66.8%); however, they were mostly male in Gambia (71.4%), Guinea (52.6%), and Malawi (70%). In our database, the SARS-CoV-2-infected HCWs were mostly hospitalized in 2020 in three countries, namely Gambia (71.4%), Ghana (85.9%), and Guinea (53.9%). The overall duration of hospitalization in the five countries was less than 30 days for most patients (96%) with a median duration of 7 days (range 4 to 11) ( Table 1).

**Table 1.** Demographic and clinical characteristics of healthcare workers hospitalized for COVID-19 stratified by country in five Sub-Saharan African countries from January 2020 to December 2022.

Countries	Gambia	Ghana	Guinea	Malawi	South Africa	Overall
Characteristics	n=7 (%)	n=99 (%)	n=76 (%)	n=10 (%)	n=11229 (%)	N=11421 (%)
**Age in years**						
<39	3 (42.9)	67 (67.7)	39 (51.3)	1 (10.0)	2898 (25.8)	3008 (26.3)
40-49	2 (28.6)	13 (13.1)	11 (14.5)	5 (50.0)	2614 (23.3)	2645 (23.2)
50 and over	2 (28.6)	19 (19.2)	26 (34.2)	4 (40.0)	5717 (50.9)	5768 (50.5)
**Median age (IQR) in years**	40.0 (33.0, 50.0)	34.0 (28.0, 45.5)	39.0 (30.0, 55.0)	46.5 (41.0, 53.8)	50.0 (39.0, 59.0)	50.0 (39.0, 59.0)
**Gender**						
Female	2 (28.6)	60 (60.6)	36 (47.4)	3 (30.0)	7526 (67.0)	7627 (66.8)
Male	5 (71.4)	39 (39.4)	40 (52.6)	7 (70.0)	3702 (33.0)	3793 (33.2)
**Year of hospital admission**						
2020	5 (71.4)	85 (85.9)	41 (53.9)	3 (30.0)	5910 (52.6)	6044 (52.9)
2021	2 (28.6)	14 (14.1)	35 (46.1)	4 (40.0)	4582 (40.8)	4637 (40.6)
2022	0 (0.0)	0 (0.0)	0 (0.0)	3 (30.0)	737 (6.6)	740 (6.5)
**Diagnosis**						
PCR confirmed	7 (100.0)	99 (100.0)	76 (100.0)	10 (100.0)	10890 (97.0)	11082 (97.0)
Clinically diagnosed	0 (0.0)	0 (0.0)	0 (0.0)	0 (0.0)	339 (3.0)	339 (3.0)
**Duration of hospitalization in days**						
30 days or less	6 (100.0)	91 (98.9)	70 (100.0)	10 (100.0)	10733 (96.0)	10910 (96.0)
Over 30 days	0 (0.0)	1 (1.1)	0 (0.0)	0 (0.0)	452 (4.0)	453 (4.0)
**Median duration of hospitalization (IQR)**	6.50 (6.00, 7.00)	10.0 (2.00, 17.0)	9.00 (8.00, 10.8)	10.0 (8.00, 10.8)	7.00 (4.00, 11.0)	7.00 (4.00, 11.0)

IQR: Interquartile Range; PCR: Polymerase Chain Reaction.

Table 2 presents the comorbidities of patients. In all five countries, a minority of patients (between 1.1% and 37.5%) had comorbidities, such as chronic cardiac disease, hypertension, asthma, chronic pulmonary disease, chronic kidney disease, HIV, cancer and tuberculosis. The most common comorbidities in the overall study population were hypertension (37%) followed by diabetes mellitus (18.5%), asthma (6.1%) and HIV-infection (4.7%) ( Table 2).

**Table 2.** Treatment comorbidities among healthcare workers hospitalized for COVID-19 stratified by country in five countries in Sub-Saharan Africa between January 2020 to December 2022.

Countries	Gambia	Ghana	Guinea	Malawi	South Africa	Overall
Comorbidities	n=7 (%)	n=99 (%)	n=76 (%)	n=10 (%)	n=11229 (%)	N=11421 (%)
**Chronic cardiac disease**						
No	6 (100.0)	87 (98.9)	75 (98.7)	6 (66.7)	8955 (98.0)	9129 (98.0)
Yes	0 (0.0)	1 (1.1)	1 (1.3)	3 (33.3)	182 (2.0)	187 (2.0)
**Hypertension**						
No	4 (80.0)	72 (81.8)	62 (81.6)	3 (50.0)	5867 (62.6)	6008 (63.0)
Yes	1 (20.0)	16 (18.2)	14 (18.4)	3 (50.0)	3498 (37.4)	3532 (37.0)
**Asthma**						
No	6 (100.0)	86 (98.9)	74 (97.4)	5 (62.5)	8629 (93.8)	8800 (93.9)
Yes	0 (0.0)	1 (1.1)	2 (2.6)	3 (37.5)	566 (6.2)	572 (6.1)
**Chronic pulmonary disease**						
No	6 (100.0)	87 (98.9)	75 (98.7)	8 (100.0)	8967 (98.5)	9143 (98.5)
Yes	0 (0.0)	1 (1.1)	1 (1.3)	0 (0.0)	139 (1.5)	141 (1.5)
**Chronic kidney disease**						
No	5 (100.0)	87 (100.0)	76 (100.0)	8 (100.0)	9063 (99.4)	9239 (99.4)
Yes	0 (0.0)	0 (0.0)	0 (0.0)	0 (0.0)	55 (0.6)	55 (0.6)
**Tuberculosis**						
No	7 (100.0)	NA	76 (100.0)	8 (100.0)	9024 (98.6)	9115 (98.6)
Yes	0 (0.0)	NA	0 (0.0)	0 (0.0)	130 (1.4)	130 (1.4)
**HIV**						
No	7 (100.0)	87 (100.0)	76 (100.0)	6 (75.0)	8670 (95.2)	8846 (95.3)
Yes	0 (0.0)	0 (0.0)	0 (0.0)	2 (25.0)	439 (4.8)	441 (4.7)
**Cancer**						
No	7 (100.0)	86 (98.9)	76 (100.0)	8 (100.0)	9041 (99.5)	9218 (99.5)
Yes	0 (0.0)	1 (1.1)	0 (0.0)	0 (0.0)	49 (0.5)	50 (0.5)
**Diabetes mellitus (any type)**						
No	0 (0.0)	95 (96.0)	69 (91.0)	8 (81.8)	9282 (81.4)	9459 (81.5)
Yes	7 (100.0)	4 (4.0)	7 (9.0)	2 (18.2)	2124 (18.6)	2148 (18.5)
**Obesity**						
No	7 (100.0)	87 (100.0)	74 (97.4)	10 (100.0)	10636 (94.8)	10635 (94.9)
Yes	0 (0.0)	0 (0.0)	2 (2.6)	0 (0.0)	593 (5.2)	594 (5.1)

NA: Not Available; HIV: Human Immunodeficiency Virus.

### Treatment received

Table 3 shows the treatments received by patients and their exit outcomes. The medical treatments most commonly used in 3/5 countries were antibiotics, (80%); they were used less often in South Africa (7.8%) and not administered in the Gambia. In Ghana, the main medical treatments were antibiotics (67.8%) followed by antimalarials (30.4%). In Guinea, the main medical treatments were antibiotics (97.3%), antimalarials (87.8%), and corticosteroids (12.5%). In Malawi, the main medical treatments were antibiotics (80%), corticosteroids (60%), and non-steroidal anti-inflammatory and antirheumatic products (50%). In South Africa, the main medical treatments were antibiotics (7.8%) and corticosteroids (7.4%).

**Table 3.** Type of medical treatment received and exit outcomes among healthcare workers hospitalized for COVID-19 stratified by country in five countries in Sub-Saharan Africa between January 2020 to December 2022.

Countries	Gambia	Ghana	Guinea	Malawi	South Africa	Overall
Type of treatment	n=7 (%)	n=99 (%)	n=76 (%)	n=10 (%)	n=11229 (%)	N=11421 (%)
**Agents acting on renin-angiotensin system**						
No	5 (100.0)	73 (92.4.0)	32 (100.0)	NA	11167 (99.5)	11277 (99.4)
Yes	0 (0.0)	6 (7.6.0)	0 (0.0)	NA	60 (0.5)	66 (0.6)
**Antibiotics**						
No	7 (100.0)	29 (32.2)	2 (2.7)	2 (20.0)	10358 (92.2)	10398 (91.1)
Yes	0 (0.0)	61 (67.8)	73 (97.3)	8 (80.0)	871 (7.8)	1013 (8.9)
**Anti-inflammatory and antirheumatic products (excl. steroids)**						
No	7 (100.0)	73 (93.6)	31 (96.9)	3 (50.0)	11075 (98.6)	11189 (98.6)
Yes	0 (0.0)	5 (6.4)	1 (3.1)	3 (50.0)	152 (1.4)	161 (1.4)
**Antimalarials**						
No	7 (100.0)	55 (69.6)	9 (12.2)	10 (100.0)	3204 (99.2)	3285 (96.6)
Yes	0 (0.0)	24 (30.4)	65 (87.8)	0 (0.0)	26 (0.8)	115 (3.4)
**Antimycotics**						
No	7 (100.0)	77 (98.7)	32 (100.0)	9 (90.0)	11215 (99.9)	11340 (99.9)
Yes	0 (0.0)	1 (1.3)	0 (0.0)	1 (10.0)	14 (0.1)	16 (0.1)
**Antivirals**						
No	7 (100.0)	78 (97.5)	29 (90.6)	8 (80.0)	10998 (97.9)	11120 (97.9)
Yes	0 (0.0)	2 (2.5)	3 (9.4)	2 (20.0)	231 (2.1)	238 (2.1)
**Corticosteroids**						
No	7 (100.0)	76 (97.4)	28 (87.5)	4 (40.0)	10400 (92.6)	10515 (92.6)
Yes	0 (0.0)	2 (2.6)	4 (12.5)	6 (60.0)	829 (7.4)	841 (7.4)
**Required ICU admission**						
No	7 (100.0)	59 (96.7)	74 (97.4)	10 (100.0)	9771 (87.0)	9920 (87.1)
Yes	0 (0.0)	2 (3.3)	2 (2.6)	0 (0.0)	1458 (13.0)	1463 (12.9)
**Died**						
No	7 (100.0)	99 (100.0)	71 (93.4)	10 (100.0)	9828 (87.5)	10015 (87.7)
Yes	0 (0.0)	0 (0.0)	5 (6.6)	0 (0.0)	1401 (12.5)	1406 (12.3)

NA: Not Applicable; ICU: Intensive Care Unit.

### Case fatality rate and effects of demographic characteristics and comorbidities on mortality among HCWs

**Figure 2.**
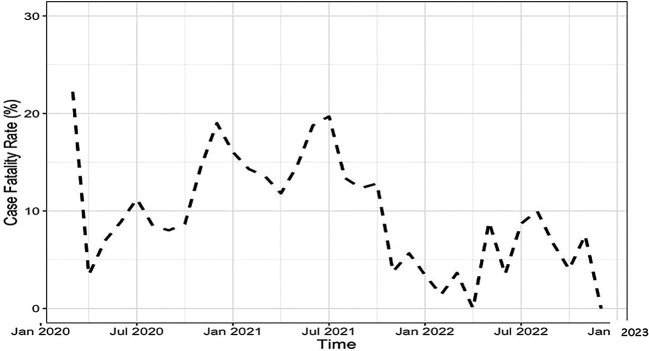
Case fatality rate over time among HCWs hospitalized for COVID-19 in Guinea and South Africa (combined) from January 2020 to December 2022.

Deaths were recorded in two out of the five countries, namely Guinea (CFR: 6.6%) and South Africa (CFR: 12.5%) ( Table 3). Figure 2 highlights the combined CFR among patients in Guinea and South Africa over time. Overall, CFR varied between 22% in March 2020 and 1.4% in February 2022, with sharp increases in December 2020 (19%) and July 2021 (19%).

**Figure 3.**
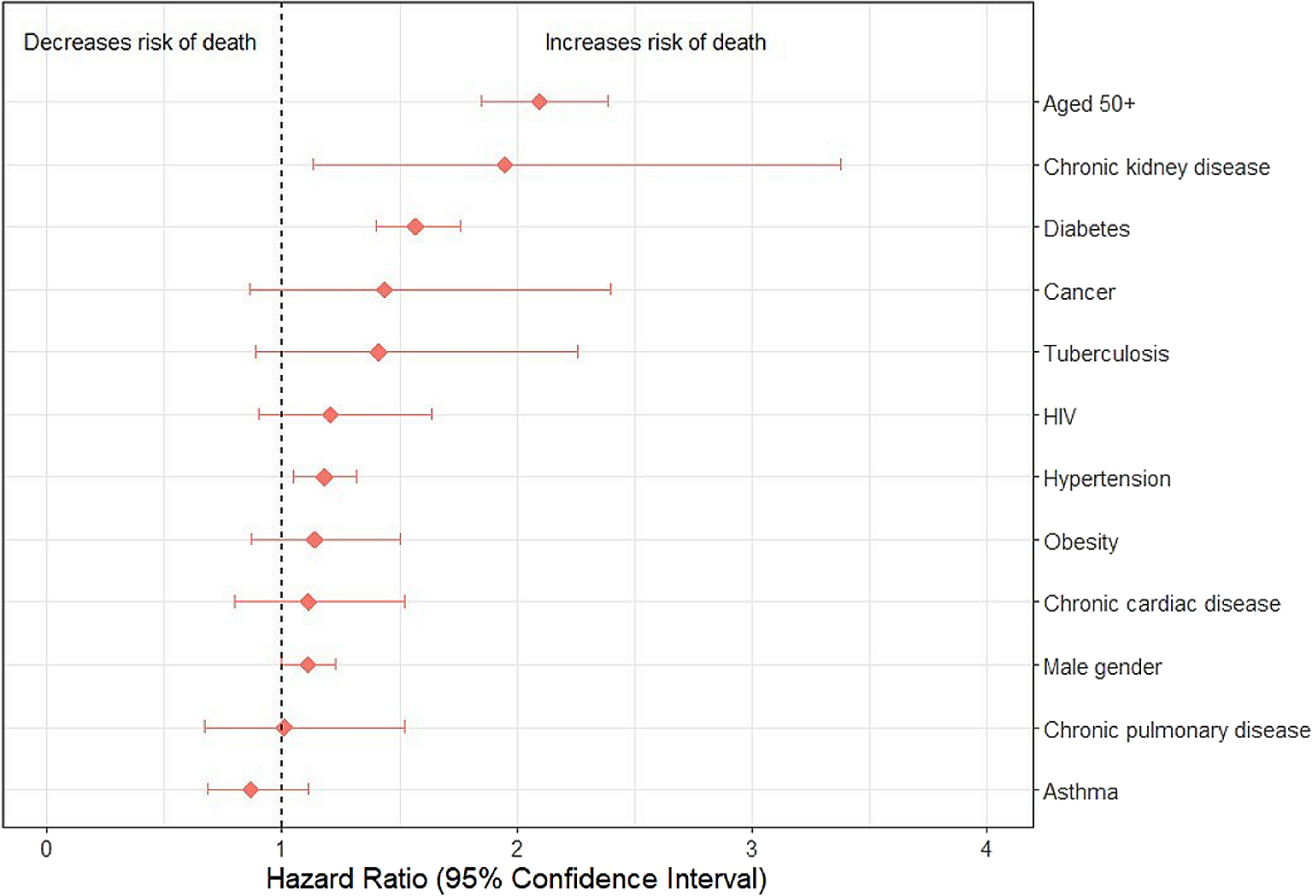
Effect of demographic characteristics and comorbidities on mortality among HCWs hospitalized for COVID-19 in Guinea and South Africa (combined) from January 2020 to December 2022.

Figure 3 depicts the associations of age, gender, and several comorbidities with risk of death among HCWs with COVID-19. The following characteristics were significantly associated with an increased risk of death among hospitalized HCWs: age over 50 years [HR 2.1 (1.85, 2.39)], hypertension [1.18 (1.05, 1.32)], diabetes mellitus [1.57 (1.4, 1.76)] and chronic kidney disease [1.95 (1.13, 3.38)].

## Discussion

Our study contributes to the limited body of research examining the clinical characteristics, case management, and health outcomes of HCWs who were hospitalized after being infected with SARS-CoV-2 in Sub-Saharan Africa. The study enrolled patients from five sub-Saharan countries (Guinea, Gambia, Ghana, Malawi, and South Africa).

We found that South Africa recorded a lower percentage (2.4%) of patients who were HCWs compared to the other four countries, likely due to the nature of the South African data collection as a national hospital surveillance study, in contrast to the more focused, higher tier care facilities recruiting patients in the other countries. Overall, HCWs hospitalized with COVID-19 were mostly over 50 years and female. In 3 out of 5 countries, however, the majority were younger (Gambia, Guinea, and Ghana) and male (Gambia, Guinea, and Malawi); antibiotics were the most commonly used medical treatments across three countries (Ghana, Guinea, and Malawi), while antimalarials were prevalent in Guinea and Ghana. Guinea and South Africa reported deaths with CFR varying between 22% in March 2020 and 1.4% in February 2022 and risk factors associated with increased mortality included age over 50 years, hypertension, diabetes mellitus, and chronic kidney disease.

In addition to the sampling and recruitment methods, several other factors could have contributed to the lower rate of HCWs affected by COVID-19 in South Africa compared to the other four countries. For example, there may have been more stringent infection control measures in healthcare settings, including adequate provision of personal protective equipment (PPE), appropriate training on infection prevention and control, strong adherence to safety measures, and access to healthcare infrastructure and resources, including hospital capacity, staffing levels, and access to resources. Many studies have already reported on these factors playing a role in preventing HCWs from SARS-CoV-2 infection. ^17^^,^^24^^–^^28^ However, Guinea has experienced previous epidemics (e.g., Ebola virus disease in 2013-2016 and 2022) and learnt from them ^29^; thus, the proportion of HWCs among COVID-19 patients may be expected to be lower, bringing it closer to that of South Africa. The experience of South Africa and Guinea with preparedness for the Ebola epidemic ^30^^–^^32^ might have also played a role in the observed findings. Nevertheless, the observed situation can be complex and influenced by various interrelated factors, including public health interventions, healthcare system capacity, and the overall epidemiological landscape of each country. Thus, additional research would be needed to provide a comprehensive understanding of why South Africa had a lower percentage of HCWs contracting COVID-19 compared to data collected in other countries.

Overall, patients were predominantly older and female. This predominance of infection among older HCWs might be related to their higher exposure to infected patients because of their experience and seniority, ^25^^,^^33^ particularly those working in high-risk settings such as inpatient and nursing homes. ^12^^,^^34^ When it comes to gender, **higher representation of women in frontline role**s, especially in patient-centric roles, such as nursing ^12^^,^^34^ and maternal healthcare, ^35^ might play a role in them being more exposed and getting more infected with SARS-CoV-2. In both older and female HCWs, the increased prevalence of infection might also be accentuated by the lack of training on infection and prevention control, ^36^ and l **imited access to PPE or** inconsistent use of PPE or improperly-fitted PPE. ^12^^,^^25^^,^^33^^,^^34^

Our findings align with those of a worldwide systematic review on infection and mortality of HCWs from COVID-19 which revealed that infections occurred mostly in women (71.6%, n=14,058) and older individuals. ^37^ Similarly, our results are supported by a first report from a living systematic review and meta-analysis on risk factors for COVID-19 infection among HCWs. ^38^ The review showed that female HCWs were at 11% higher risk of COVID-19 than their male counterparts, but it failed to provide underlying reasons and mechanisms. Therefore, further studies addressing sex and gender disparities in COVID-19 infection among HCWs are encouraged to generate evidence-based recommendations for decision-making. In contrast, in the general population, the existing literature on sex and gender differences in COVID-19 infection shows a higher risk of severe infection in men compared with women. ^39^^–^^41^

Overall, the CFR fluctuated between 22% in March 2020 and 1.4% in February 2022. Notably, a sharp rise in CFR was observed in December 2020 (19%) and July 2021 (19%). These increases overlapped with the second wave of the pandemic in South Africa, which was characterized by the prevalence of the Beta variant which dominated new infections between October 18, 2020, to April 30, 2021. ^42^ Our analysis suggests that HCWs faced increased challenges related to the severity of the pandemic within the broader population, highlighting their vulnerability and the need for improved resilience and adaptive responses. Consequently, health systems must learn from their experiences (and from others’ experiences) during the COVID-19 pandemic and enhance their preparedness and responsiveness for future health crises. ^29^

We found that being over 50 years of age, being male, and having certain comorbidities was associated with a higher risk of death among HCWs in Guinea and South Africa. Overall, these results corroborate the existing literature on risk factors for mortality in COVID-19 patients in sub-Saharan Africa, ^43^ specifically in Guinea ^44^^,^^45^ and in South Africa. ^46^ However, our findings are contrary to those of a previous study regarding gender in South Africa; this study revealed that patients with a higher risk of death were mostly of female gender (beyond being aged 60 years or older and having comorbidities). However, that South African study only dealt with deaths within the first 24 hours. ^47^ Hence, additional research is required to elucidate gender discrepancies concerning the risk of mortality among COVID-19 patients in South Africa.

Even though COVID-19 infection is a viral infection, we observed a higher usage of antibiotics among HCWs, except in South Africa. Similar findings were seen in a study conducted in Sierra Leone among suspected and confirmed COVID-19 patients admitted to isolation units and treatment centers. ^48^ This suggests that both HCWs and non-HCWs were given antibiotics in the management of viral infection. This calls for effective implementation and supervision of case management guidelines during infectious disease outbreaks as all national and international guidelines did not recommend widespread use of antibiotics.

### Limitations

This study is subject to certain limitations. The main limitation is that the sample sizes of all countries except South Africa are very limited, restricting our study to descriptive analyses. Given the small sample sizes, we were thus unable to delineate specific patterns on a country-by-country basis, such as CFR. Disease classification used did not allow us to ascertain COVID-19 disease severity among HCWs. We reported exit outcomes for only two countries.

## Conclusions

The results of our study underscore the necessity for continuous support of HCWs, especially during epidemics and pandemics. Similarly, they emphasize the importance of enhancing countries' readiness for health crises and improving Infection Prevention Control measures among HCWs, with a particular focus on older and female individuals. Additional research would be needed to provide a comprehensive understanding of the findings of this study.

## Data Availability

All individual patient data are available for access. Researchers may request access to data by submitting an application ( https://www.iddo.org/document/covid-19-data-access-application-form) to covid-19@iddo.org. Decisions on all applications are made by an independent Data Access Committee who review applications for compliance with the Data Access Guidelines of the COVID-19 platform available at https://www.iddo.org/document/iddo-data-access-guidelines . Requirements include scientific value and validity, plans for collaboration and knowledge sharing, a lack of ethical concerns, and researcher status to deliver the work. All approved applications are publicly available ( https://www.iddo.org/covid19/research/approved-uses-platform-data ).
