# Supplementary material for: Epidemiological profiles and outcomes of healthcare workers hospitalized for COVID-19 in five Sub-Saharan African countries: a cohort study
Source: F1000Res. 2024 Jun 18;13:655. [Version 1] doi: 10.12688/f1000research.150775.1 (PMC12238847; doi:10.12688/f1000research.150775.1)
Supplement: Peer review history

# Reviewer report: Reviewer response for version 1

**DOI:** 10.5256/f1000research.165373.r385706

**Published:** 2025-07-08

**Authors:** Carla Barros

**Affiliations:** 1 Universidade Fernando Pessoa, Porto, Porto District, Portugal

This study aimed to analyze the epidemiological profiles and outcomes of HCWs hospitalized for COVID-19.

 The manuscript is methodologically highly relevant.

 Improvements can be made in (i) framing, interpretation, and clarity highlighting its contribution to the global COVID-19 literature and health systems and (ii) expanding the conclusions with a deeper analysis.

Is the work clearly and accurately presented and does it cite the current literature?

Yes

If applicable, is the statistical analysis and its interpretation appropriate?

Yes

Are all the source data underlying the results available to ensure full reproducibility?

Yes

Is the study design appropriate and is the work technically sound?

Yes

Are the conclusions drawn adequately supported by the results?

Partly

Are sufficient details of methods and analysis provided to allow replication by others?

Yes

Reviewer Expertise:

Occupational Health Psychology

I confirm that I have read this submission and believe that I have an appropriate level of expertise to confirm that it is of an acceptable scientific standard, however I have significant reservations, as outlined above.

# Reviewer report: Reviewer response for version 1

**DOI:** 10.5256/f1000research.165373.r388109

**Published:** 2025-06-24

**Authors:** Sumant Kumar

**Affiliations:** 1 Alliance University, Bengaluru, Karnataka, India

The paper explores a significant gap in healthcare worker outcomes during the COVID-19 pandemic in the Sub-Saharan Africa region. The use of ISARIC-WHO for data is a reliable source for all five selected countries. However, if the author can give more details about each country's HCW data, for instance, whether the data is national or site-specific, they should also explain the limitations of the selected sample sizes.

 Clinical severity measures need to be included the check the availability of oxygen therapy, ICU facilities and other respiratory supports as key severity markers.

 What are the measures taken to prevent infection, such as policy, information, training, etc?

 Paper should also reflect on why CFR increased in December 2020 and July 2021, including exploring whether the higher female HCWs hospitalisation is associated with differential exposure risk or due to workforce distribution.

Is the work clearly and accurately presented and does it cite the current literature?

Yes

If applicable, is the statistical analysis and its interpretation appropriate?

I cannot comment. A qualified statistician is required.

Are all the source data underlying the results available to ensure full reproducibility?

Partly

Is the study design appropriate and is the work technically sound?

Yes

Are the conclusions drawn adequately supported by the results?

Partly

Are sufficient details of methods and analysis provided to allow replication by others?

Yes

Reviewer Expertise:

Social Science and Humanities

I confirm that I have read this submission and believe that I have an appropriate level of expertise to confirm that it is of an acceptable scientific standard.

# Reviewer report: Reviewer response for version 1

**DOI:** 10.5256/f1000research.165373.r388105

**Published:** 2025-06-24

**Authors:** Mohammad Sidiq, Jyoti Sharma

**Affiliations:** 1 Galgotias University, Greater Noida, Uttar Pradesh, India; 2 Physiotherapy, SAHS, Galgotias University, Greater Noida, India

**1. Title and Abstract**

  The Title is concise, and it represents the study well

 Well-structured. Findings are well-defined and placed under clear sections on background, methods, results, and conclusions.

 Abstracts may be supplemented by quantitative information, which may be achieved with confidence intervals, to assist with statistical rigor.

**2. Introduction and Background**

- **Rationale: Justified.** There is a definite knowledge gap regarding the issue of COVID-19 among HCWs in sub-Saharan Africa.
- Contextualization: Makes proper references to the previous work and discusses peculiarities of regional vulnerabilities.
- Suggestion: Explicate the reasons behind selecting the countries that are not based on the availability of data specifically—state health system diversity or policies in curbing the pandemic.

**Methods**

- Design: Correct retrospective cohort design of secondary ISARIC information.
- Data Sources: Well defined using the ISARIC-WHO standardized forms and REDCap/Trifacta tools.
- Ethical Considerations: It is well covered, and its ethical approval has numerous levels.
- Recommendation: The inclusion of a flow diagram (e.g., PRISMA-style) of HCWs by country would be beneficial to transparency.
- Needs clarification: Did they measure the severity of the disease, or was the only proxy ICU admission?

**Results**

- Presentation: Disaggregated by country, and data are wholesome.
- Figure/Table used: They are quite sufficient, in particular Figs. 2 and 3 (CFR trends and risk ratios).
- Statistical Analysis: Cox regression is suited for investigation of risk of mortality.
- Major Concern: Sample sizes of subgroups (e.g., Malawi, Gambia) are extremely small—reduces the ability to generalize and statistical conclusions.

**Discussion**

- Interpretation: Cogitative, takes opportunity factors that include experience of prior epidemics and availability of PPE into consideration.
- Literature Integration: Cites the pertinent global and regional studies.
- Recommendation: Elaborate more on the gender disparity in terms of infections and deaths as it is demixed in different countries.
- Limitation Acknowledgment: Good, except that the effect of misclassification bias (e.g., clinically suspected cases) ought to be mentioned.

**7. References**

**Appropriate and Comprehensive:** Citations span WHO, peer-reviewed literature, and systematic reviews.

**Minor Suggestion:** Ensure reference formatting consistency, particularly with DOIs and PubMed IDs.

**10. Overall Recommendation**

**Approved with Reservations**

 This is an important and topical contribution. Although the study is sound on methodologies and contributes to an urgent gap, its interpretation is constrained by small numbers in most countries. The scientific rigor of the discussion could also be enhanced by enhancing arguments of limitations and potentially clarifying abstract and methods.

Is the work clearly and accurately presented and does it cite the current literature?

Yes

If applicable, is the statistical analysis and its interpretation appropriate?

Partly

Are all the source data underlying the results available to ensure full reproducibility?

Yes

Is the study design appropriate and is the work technically sound?

Yes

Are the conclusions drawn adequately supported by the results?

Yes

Are sufficient details of methods and analysis provided to allow replication by others?

Yes

Reviewer Expertise:

Musculoskeletal, Public Health, Clinical Research, Image Processing, and Pain sciences

We confirm that we have read this submission and believe that we have an appropriate level of expertise to confirm that it is of an acceptable scientific standard, however we have significant reservations, as outlined above.
